# Clinical effect of peripherally inserted central catheters based on modified seldinger technique under guidance of vascular ultrasound

**DOI:** 10.12669/pjms.325.10384

**Published:** 2016

**Authors:** Qingguo Wang, Ni Wang, Yuzhen Sun

**Affiliations:** 1 Qingguo Wang, Department of Special Inspection, Binzhou People’s Hospital, Shandong 256603, China; 2 Ni Wang, Department of Special Inspection, Binzhou People’s Hospital, Shandong 256603, China; 3 Yuzhen Sun, Department of Neurosurgery, Binzhou People’s Hospital, Shandong 256603, China

**Keywords:** Vascular Ultrasound, MST, PICC, Effect of Catheterization

## Abstract

**Objective:**

To observe and analyze the application effect of ultrasound-guided modified Seldinger technique (MST) in Peripherally Inserted Central Catheter (PICC) catheterization.

**Methods:**

Two hundred patients treated with PICC catheterization from January 2013 to December 2015 were selected and randomly divided into two groups, namely, observation group and control group. The observation group adopted ultrasound-guided MST for catheterization while the control group applied traditional puncture technique for catheterization. Then efficacy of catheterization, success rate of catheterization and incidence rates of complications were compared between two groups.

**Results:**

Various indicators of catheterization effects of the observation group were better than those of the control group, and the differences were statistically significant (P<0.05); one-time success rate of puncture and catheterization of the observation group was both higher than the control group (P<0.05);. Moreover, the incidence of puncture points bleeding, phlebitis and thrombus were all lower than those of the control group (P<0.05).

**Conclusion:**

Implementing PICC catheterization based on ultrasound-guided modified Seldinger puncture technique can increase success rate of puncture, improve the effect of catheterization, lower incidence rate of adverse effects of catheterization and improve satisfaction and comfort level of patients.

## INTRODUCTION

Peripherally Inserted Central Catheter (PICC) makes quick dilution of intravenous dripping drugs possible and is capable of reducing stimulus of drugs to blood vessels. Also, it can prevent patients from suffering from pain induced by repeated puncture and intensely irritant drugs. For long-term venous transfusion, and process of intermittent treatment, PICC is recognized as a safe, economical and reliable venous transfusion way, which causes little trauma, and it is widely used in clinical practices.^1^,^2^ However, due to obesity and poor conditions of veins of some patients, success rate of PICC puncture is low, and it is likely to cause complications such as phlebitis, which obviously shorten time of continuous using of PICC catheter.^3^,^4^

In China, vessel puncture is a conventional puncture technique of PICC embedding, which requires thick, straight, and elastic blood vessels. For those patients who are in urgent need of catheterization but with poor vein conditions, traditional PICC puncture has disadvantages for it is time consuming, and might cause great damage to local tissues.^5^,^6^ In 1997, Claudette Boudreaux, a nurse of intensive care unit (ICU) in medical center of Washington, used Modified Seldinger Technique (MST) to do PICC puncture on patients under the guidance of ultrasound, which raised the success rate of bedside PICC cathetering from 65% to 91%.^7^ Using ultrasound-guided Modified Seldinger Puncture Technique to implement PICC catheterization can accurately place catheters into veins under the monitoring of ultrasound, which obviously increases puncture rate and success rate of catheterization, and reduces complications caused by puncture.^8^ The study adopted ultrasound-guided MST in PICC catheterization, and obtained good results. The report is as follows.

## METHODS

### General data

Two hundred patients who were treated with PICC catheterization in Binzhou People’s Hospital from Jan. 2013 to Dec. 2015 were selected as research objects. Patients who had venous transfusion time≥6 d, have underwent chemotherapy, were conscious with good cognitive abilities, were aware of research contents and volunteered to sign informed consent form were included. They were divided into two groups, observation group (N=100) and control group (N=100), according to random number table. There were 60 males and 40 females in the observation group, aged from 20 to 69 years (average (52.1±3.4) years), among which there were 42 cases of lung cancer, 33 cases of breast cancer, 19 cases of stomach cancer, and 6 cases of liver cancer. In the control group, there were 63 males and 37 females, aged from 21 to 70 years (average (53.1±3.7) years of age). It included 40 cases of lung cancer, 32 cases of breast cancer, 23 cases of stomach cancer and 5 cases of liver cancer. Differences of general data between of two groups were not statistically significant (P>0.05); hence the results were comparable.

### The control group

Traditional puncture method (blind puncture) was adopted: operators evaluated blood vessels of lower arm and marked the puncture points through visual inspection, touch or according to clinical experiences of puncture. The operator used left hand to fix skin of puncture point, and used right hand to insert needle into blood vessels of puncture point at the angle of 30° by right hand using 16G steel puncture needles. When saw the blood returned, the operator pulled out the needle and put the catheter to expected location by puncture sheath.

### The observation group

Ultrasound-guided MST puncture method was adopted: We selected GE vivid e9 ultrasound equipment and Seldinger puncture suite produced by General Electric Company. Patients were in horizontal position, and the catheterized limbs extended at the angle of 60° to 90°. Then we used vascular ultrasound instruments to select proper veins above elbow and mark them. And we measured the length of catheters that were to be inserted, routinely sterilized the catheterization parts, put sterile cover over probe and smeared sterilizing coupling agents. The assistant tied tourniquet, while the operator inserted puncture needle into blood vessels from marked puncture points. After blood returned, the operator used color doppler ultrasound to observe whether blood vessels were unobstructed and pushed into outer cannulas and polled out corn of needle. Next, the guiding wire was pushed for 5-10 cm with tourniquet tied. If the delivery was unhindered, the tourniquet was untied and the guiding wire was pushed for 15 cm. After outer cannulas were drawn out, the puncture point was covered with gauze. Lidocaine was injected subcutaneously around puncture points, and needle eye was pressed with gauze for 5-10 s. Then the truncated edge of skin expander was cling to tip of guiding wire and was put into hypoderm for 0.3-0.5 cm, to expand the incision. Next, the vascular dilator and leading-in sheath components were put to skin incision through the end tail of guiding wire, and the leading-in sheath was put into the incision. After conforming by ultrasound that the leading-in sheath had entered into a blood vessel, the dilator and leading-in sheath were separated, and the dilator and the guiding wire were taken out. After ultrasound suggested PICC catheters had entered into veins, the supporting guiding wire was taken out, and was routinely fixed. At last, the location of the tip of the catheter was identified using X-ray.

### Observation Index

(1) Effects of catheterization: this indicator mainly included achievement of expected goals, comfort levels, pain score, and rate of puncture failure; (2) Success rate of catheterization: this indicator mainly included success rate of puncture (puncture at one time as success, repeated puncture as failure) and success rate of catheterization (puncture at one time as success, repeated puncture as failure); (3) Incidence rates of complications: this indicator mainly included incidences rates of puncture site bleeding, phlebitis, and thrombus 2 months after puncture.

### Determination criteria of curative effect

The first criterion was comfort level. Comfort level questionnaire developed by our hospital was used for investigation. The questionnaire was designed by selecting 10 issues which was concerned most by patients based on literature. The score of each item was 1 ~ 5 points. A total score of 45 ~ 50 points was determined as comfort, 25 ~ 34 points as general, 15 ~ 24 points as discomfort, and less than 15 points as quite uncomfort, i.e., higher score meant higher comfort level. The questionnaire was evaluted with a content validity of 0.81 and 0.856 Cronbach’α coeffcient by five clinical experts, suggesting its good reliability and validity. The second criterion was pain score. The degree of pain was scored using numerical rating scale. A straight line with a length of 10 cm was divided into 10 pieces evenly, marked as 0 ~ 10 points respectively. Patients marked the line by himseves according to the pain they suffer. Higher score meant more serious pain. 0 point stands for no pain, 1 ~ 3 points stands for mild pain, 4 ~ 6 points as medium pain, and higher than 7 points as severe pain.

### Statistical methods

All data were analyzed by SPSS 20.0 statistical software, and the measurement data were expressed by mean±SD. The comparisons were tested by using t-test, while the enumeration data were expressed by percentage and the comparisons were tested by chi-square test. If P<0.05, the differences were considered as statistically significant.

## RESULTS

### Catheterization effect of patients in two groups

Various indicators of catheterization effects of two groups of the observation group were better than those of the control group. Among the observation group, there were 93 cases whose treatment effects met expectation while in the control group, there were only 64 cases. Failure rate of the observation group was only 2.0%, which was obviously lower than the control group (34%); Pain score of the observation group was lower than that of the control group, and the comfort levels score of the former was higher than the latter. The differences of the indicators between two groups were statistically significant (P<0.05). Table-I.

**Table-I:** Comparison of catheterization effects of patients.

*Group*	*Achieve expected goal (%)*	*Failure rate of puncture (%)*	*Pain score*	*Comfort levels*
Observation Group (N=100)	93(93.0)	2(2.0)	2.61±0.95	47.24±1.42
Control Group (N=100)	64(64.0)	34(34.0)	5.21±1.49	36.35±2.23
X^2^/t value	1.622	14.853	12.252	4.022
P value	<0.05	<0.05	<0.05	<0.05

*Note:* The full mark of comfort levels was 50, the higher the score, the higher the comfort levels; the full mark of pain score was 10, the higher the scores, the greater the pain degree.

### Success rate of catheterization of two groups

One-time success rate of puncture and one-time success rate of catheterization of the observation group were higher than those of the control group, and the differences were statistically significant (P<0.05). Table-II.

**Table-II:** Comparison of one-time success rate of puncture and catheterization of patients [N(%)].

*Group*	*One-time success rate of puncture*	*One-time success rate of catheterization*
Observation Group (N=100)	98(98.0)	100(100.0)
Control Group (N=100)	81(81.0)	80(80.0)
X2	6.135	4.051
P value	<0.05	<0.05

### Occurrence of complications of two groups

The incidence of puncture points bleeding, phlebitis and thrombus of the observation group was all lower than those of the control group (P<0.05). Table-III.

**Table-III:** Comparison of incidence rates of complications of patients [N(%)].

*Group*	*Puncture points bleeding*	*Phlebitis*	*Thrombosis*
Observation Group (N=100)	5(5.0)	2(1.0)	0(0.0)
Control Group (N=100)	17(17.0)	8(8.0)	6(6.0)
X^2^	12.468	3.376	6.970
P value	<0.05	<0.05	<0.05

### Comparison of success rate of catheterization

As to the observation group, the one-time, two-time, three or higher-time success rate of catheterization of patients with 2.0 ~ 2.5 mm vescular diameter was 20.0%, 60.0% and 20.0% respectively; the success rate of patients with 2.6 ~ 3.0 mm vascular diameter was 85.7%, 9.5% and 4.8% respectively; the success rate of patients with 3.1 ~ 4.0 mm vascular diameter was 91.7%, 8.3% and 0; the success rate of patients with 4.0 mm larger vascular diameter was 100%, 0% and 0%. It indicated that, patients with larger vascular diameter had higher one-time success rate of cauterization.

## DISCUSSION

Traditional puncture methods adopt means of touching and external visual inspection. Some of the methods evaluate the conditions of blood vessels according to experience before puncture. However, for patients who are fat, with flabby skins or with poor vessel conditions, it is difficult for traditional blind puncture to achieve one-time success.^9^ Ultrasound-guided MST puncture technique is able to directly see size of diameter of punctured blood vessel, thereby enlarging views of puncture to pinpoint the puncture points. In addition, puncture needles of sheathes of capillaries are small. All these advantages overcome disadvantages of traditional puncture to some extent and significantly improve the success rate of puncture.^10^,^11^ Results of the study showed, one-time success rates of puncture and catheterization of the observation group were far higher than those of the control group; patients with larger vascular diameter had higher sucess rate.

A recent study showed that, patients who received traditional puncture methods might be required to draw out catheters because of unplanned catheters drawing, high incidence rate of complications, etc, during later period of therapeutic process with catheters, which greatly influenced the comfort levels of treatment period with catheters.^12^ But using ultrasound-guided MST puncture technique to do catheterization could effectively avoid it. The reason might be that the catheterization positions of patients of the observation group was above elbow joints, which was flat and easy for sterile films to be fixed; Patients could unrestrictedly bend and stretch their upper arms, which avoided curling up of fringes of films due to movements and sweating, thus reducing times of dressing change and film changing.^13^,^14^ Also, as time went on, patients pay more attention to the safety of catheterization and whether it could relieve the pain and successfully complete the treatment.^15^ Results of the study showed that differences of achievement of expected goals and comfort levels between two groups during the catheterization were significant; failure rate of the observation group was only 2.0%, which was obviously lower than 34% of the control group; comfort levels of the observation group was higher than the control group, and the pain score of the former was lower than the latter; indicators mentioned above showed that the effects of ultrasound-guided MST catheterization technique were significantly better than the effects of traditional PICC catheterization technique.

Moreover, results of the study showed that, incidence of puncture points bleeding and phlebitis of the observation group were lower than those of the control group. Traditional PICC catheterization puncture does great damage to vessels and leads to incidence of complications such as phlebitis and puncture points’ bleeding.^16^ MST adopting thin puncture needles can clearly demonstrate the conditions of vessels under the guidance of ultrasound; what is more, catherization above elbow can reduce the tension and friction of duct along with elbow joint movement; duct is in a floating state in blood vessels as the vessels above elbow have large diameter and fast blood flow speed, which will not affect blood circulation and can reduce incidence of puncture points bleeding and mechanical phlebitis.^17^,^18^ The study found, incidence rate of thrombus of the observation group was also lower than the control group. Phlebothrombosis is the most dangerous complication of PICC, which happens generally 14 to 53 days after catheterization. The cause of phlebothrombosis formation is closely related to damages of vascular wall.^19^ MST is capable of visually showing positions and conditions of veins, preventing mistaken puncture, penetration into vessels, repeated puncture and etc, from happening. Meanwhile it reduces damages caused by puncture to endangium and incidence rate of phlebothrombosis.

## CONCLUSION

To sum up, using MST to implement PICC catheterization under the guidance of high-definition and visual vascular ultrasound provides cancer patients with safe and effective intravenous indwelling channels. Due to the catheterization positions moving from below elbow to above elbows, applications of vascular ultrasound systems, and updates of puncture technique, occurrences of complications after PICC catheterization were greatly reduced, which ensured the success of catheterization. Compared to traditional blind puncture technique, MST is characterized by low incidence of complications during catheterization, high comfort levels, low failure rate of puncture, low pain scores, etc. Thus, it can be regarded as the preferred puncture scheme for patients who require long-term transfusions, for it is significantly effective and deserved to be clinically generalized and applied.
